# Genome Sequences of Multidrug-Resistant Salmonella enterica Serovar Dublin Isolates

**DOI:** 10.1128/MRA.00698-19

**Published:** 2019-08-01

**Authors:** Baha Abdalhamid, Emily L. Mccutchen, Kacie D. Flaherty, Steven H. Hinrichs, Peter C. Iwen

**Affiliations:** a Department of Pathology and Microbiology, University of Nebraska Medical Center, Omaha, Nebraska, USA; b Nebraska Public Health Laboratory, University of Nebraska Medical Center, Omaha, Nebraska, USA; University of Arizona

## Abstract

Salmonella enterica serovar Dublin, which can cause enteritis and systemic infections in humans, has been associated with antimicrobial resistance. Here, we report draft genome sequences of seven multidrug-resistant *S*. Dublin isolates from human samples. These sequences will contribute to an understanding of pathogenesis and resistance determinants in this serovar.

## ANNOUNCEMENT

Salmonella enterica serovar Dublin, a significant cause of foodborne diseases for animals and humans, is generally transmitted through unpasteurized milk and cheese (1, 2). It is also known to have a propensity for causing invasive infections with multidrug resistance, which complicates the therapeutic approach (1, 3–6). In this report, we present seven draft genome sequences of *S*. Dublin isolates collected from humans in Nebraska.

Blood, MacConkey, chocolate, and/or Hektoen enteric agar plates were used to culture human stool, blood, and synovial fluid specimens. Bacterial cultures were incubated overnight at 35°C. *Salmonella* species are non-lactose fermenters and H_2_S producers. A MicroScan WalkAway system (Beckman Coulter, Brea, CA) was used for identification of *Salmonella* species (Table 1). Serovar designations were made by pulsed-field gel electrophoresis and subsequently confirmed by whole-genome sequencing (WGS) using the MiSeq platform (Illumina, CA, USA). Strains from overnight cultures were tested for antimicrobial susceptibility to certain antibiotics using the disk diffusion method and interpreted using CLSI guidelines (Table 1).

**TABLE 1:** Summary characteristics of whole-genome sequencing of multidrug-resistant *Salmonella* serovar Dublin strains^*a*^

Strain	No. of contigs	Genome size (bp)	*N*_50_	GC content (%)	No. of genes	RNA genes	AMR phenotype^*b*^	Resistance genes^*c*^	GenBank accession no.	SRA accession no.^*d*^
SD_190068	92	4,923,539	405,624	52.11	4,989	114	AM, CAZ, TE, C	*bla*_CMY-2_, *bla*_TEM-1B_, *floR*, *strA*, *strB*, *sul2*, *tetA*	VACG00000000	SRR9641963
SD_180610	45	4,978,985	478,715	52.11	5,016	112	AM, CAZ, TE, C	*bla*_CMY-2_, *bla*_TEM-1B_, *floR*, *strA*, *strB*, *sul2*, *tetA*	VDCP00000000	SRR9641967
SD_180280^*e*^	50	4,979,905	494,424	52.11	5,032	115	AM, CAZ, TE, C, CIP	*bla*_CMY-2_, *bla*_TEM-1B_, *floR*, *strA*, *strB*, *sul2*, *tetA*, *gyrA* D87N	VACH00000000	SRR9641964
SD_170735^*e*^	65	5,136,349	402,292	52.11	5,179	121	AM, CAZ, TE, C, CIP	*bla*_CMY-2_, *bla*_TEM-1B_, *floR*, *strA*, *strB*, *sul2*, *tetA*, *gyrA* D87G	VACM00000000	SRR9641961
SD_170156	73	4,962,240	420,579	52.09	5,035	116	AM, CAZ, TE, C	*bla*_CMY-2_, *floR*, *strA*, *strB*, *sul2*, *tetA*	VACN00000000	SRR9641962
SD_12500	51	4,984,528	478,716	52.1	5,032	117	AM, CAZ, TE, C, SXT	*bla*_CMY2_, *bla*_TEM-1B_, *floR*, *strA*, *tetA*, *strB*, *sul2*	VACK00000000	SRR9641959
SD_7855	75	5,095,495	204,878	51.89	5,171	117	AM, TE, C	*bla*_TEM-1A_, *catA1*, *strA*, *strB*, *sul1*, *tetB*, *aadA1*, *aph(3′)-Ic*	VACJ00000000	SRR9641966

a Sequence type by MLST for all strains was sequence type 10 (ST10).

b Testing performed by the disc diffusion method. AMR, antimicrobial resistance; AM, ampicillin; CAZ, ceftazidime; TE, tetracycline; C, chloramphenicol; CIP, ciprofloxacin; SXT, trimethoprim-sulfamethoxazole.

c β-Lactam resistance genes, *bla*_CMY-2_, *bla*_TEM-1B_; chloramphenicol resistance genes, *floR* and *catA1*; fluoroquinolone resistance genes, *gyrA* mutations; aminoglycoside resistance genes, *strA*, *strB*, *aadA1*, and *aph(3′)-Ic*; tetracycline resistance genes, *tetA* and *tetB*; sulfonamide resistance genes, *sul1* and *sul2*.

d SRA, Sequence Read Archive.

e These two strains showed intermediate susceptibility to ciprofloxacin due to *gyrA* mutations.

A single colony of an overnight blood agar plate culture at 35°C was used for genomic DNA extraction using the MagNA Pure compact nucleic acid isolation kit I using the MagNA Pure compact instrument (Roche Diagnostics, IN, USA). DNA quality and quantity were determined using the NanoDrop 2000 UV-visible spectrophotometer (Fisher, MA, USA) and the Qubit 3.0 fluorometer (Invitrogen, CA, USA), respectively.

Genomic libraries were prepared using the Nextera XT library prep kit (Illumina) as instructed by the manufacturer. WGS was performed according to the manufacturer’s protocol using a 250-bp paired-end chemistry. The following parameters were used to assess the quality of the run: Phred quality score (quality score 30 > 75%), cluster density (600 to 1,300), and percentage of clusters passing filters (>80%). Default parameters were used for all bioinformatics software unless otherwise specified. The quality of the sequence reads was evaluated using FastQC 0.10. After subsequent quality trimming using Trimmomatic 0.33, the contigs of genomic sequences were *de novo* assembled using SPAdes 3.12, and contigs with a size less than 200 bp were filtered out using BBmap 38.06. The quality of the *de novo* assembled genomes were determined using QUAST 4.1 (Table 1). The draft genome of each strain was annotated using the NCBI Prokaryotic Genome Annotation Pipeline (PGAP) 4.8 (https://www.ncbi.nlm.nih.gov/genome/annotation_prok/). All bioinformatics tools used in this study except PGAP were made available through https://github.com/github.

The multilocus sequence types (MLST) were determined using MLST 2.0 as previously described (7). ResFinder 3.1 was used to identify antimicrobial resistance determinants, including acquired resistance genes and/or chromosomal mutations (8).

The average genome size of the sequenced isolates was 5,008,720 bp (range, 4,859,717 to 5,136,349 bp), the average number of contigs was 64 (range, 45 to 92), and the average *N*_50_ value was 412,175 (range, 204,878 to 494,424) (Table 1).

The collected data provides a basis for comparison with other *S*. Dublin strains and studies for epidemiology, surveillance, antimicrobial resistance, and pathogenesis.

### Data availability.

The BioProject number for these sequences is PRJNA541980. GenBank accession numbers, Sequence Read Archive (SRA) accession numbers, numbers of contigs, *N*_50_ values, numbers of RNA genes, MLSTs, susceptibilities, and genotypic resistance profiles are provided in Table 1.
